# Impact of *Wolbachia* on oxidative stress sensitivity in the parasitic wasp *Asobara japonica*

**DOI:** 10.1371/journal.pone.0175974

**Published:** 2017-04-20

**Authors:** David Monnin, Natacha Kremer, Emmanuel Desouhant, Fabrice Vavre

**Affiliations:** Université Lyon 1, CNRS, UMR 5558, Laboratoire de Biométrie et Biologie Evolutive, Villeurbanne, France; Pusan National University, REPUBLIC OF KOREA

## Abstract

The oxidative homeostasis is the balance between reactive oxygen species and antioxidant molecules. In addition to be considered as a key factor underlying life-history traits evolution, the oxidative homeostasis has been shown to be involved in many host–symbiont associations. Previous studies suggest an interaction between the bacterial endosymbiont *Wolbachia* and the oxidative homeostasis of some insect hosts. This interaction is likely to exert a strong influence on the host evolution, as it has been proposed in the wasp *Asobara tabida*, whose dependence upon *Wolbachia* is due to the evolutionary loss of its ability to regulate the oxidative homeostasis in the absence of the symbiont. Although such cases of complete dependence are rare, cases of insects having lost only a part of their autonomy over the control of the oxidative homeostasis might be more common. If so, one can expect that insects having coevolved with *Wolbachia* will be more sensitive to oxidative stress when cured of their symbionts. We tested this hypothesis by studying the effects of an experimentally-induced oxidative stress on various life-history traits of *Asobara japonica*, a species closely related to *A*. *tabida*. For most of the life-history traits studied, the sensitivity of the wasps to oxidative stress did not correlate with their infection status. The only exception was the parasitic success. However, contrarily to our expectation, the sensitivity to oxidative stress was increased, rather than decreased, when *Wolbachia* was present. This result suggests that *Wolbachia* does not participate to mitigate oxidative stress in *A*. *japonica*, and that on the contrary its presence might still be costly in stressful environments.

## Introduction

The bacterial endosymbiont *Wolbachia* is well known for its ability to invade arthropod host populations through various manipulations of reproduction, such as cytoplasmic incompatibility, male-killing, phenotypic feminisation and thelytokous parthenogenesis [1]. More recently, the impact of *Wolbachia* on host physiology has also been considered, for example to understand how *Wolbachia* mediate other phenotypes such as viral protection [2,3]. Notably, an interaction between *Wolbachia* infection and the host oxidative homeostasis has been shown in several insect species [4–9]. The oxidative homeostasis is the balance between reactive oxygen species (ROS) and antioxidant molecules. ROS are mostly produced as a by-product of the oxidative phosphorylation in mitochondria [10], but can also be produced in a controlled manner, as an immune response against microorganisms [11]. This is presumably why *Wolbachia* induces an oxidative stress–*i*.*e*., a deleterious excess of ROS relative to antioxidant molecules–in some of its hosts [4,6]. Interestingly, this *Wolbachia*-induced disruption of the oxidative homeostasis seems to be found in novel (*i*.*e*., artificially transinfected) hosts rather than in native ones [12,13]. It can therefore be hypothesized that the coevolution between the symbiotic partners leads usually to a mitigation of the *Wolbachia*-induced oxidative homeostasis disruption [13,14]. Indeed, given the strong impact of the oxidative state on the host life-history traits [15] and on the symbiotic population density [5,7,8], adaptation to *Wolbachia* is likely to involve modulations of the oxidative homeostasis.

In the parasitic wasp *Asobara tabida*, it is even thought that the evolution of mitigation led to the complete dependence of the wasp for oogenesis [16]. In aposymbiotic females (*i*.*e*., cured from *Wolbachia*), a strong apoptosis in ovaries prevents the development of fertile eggs [17]. Transcriptomic data [18,19], as well as experimental manipulation of the iron load in the food that increases oxidative stress [18], suggest that the adaptation to the presence of *Wolbachia* has resulted in the loss of *A*. *tabida* ability to regulate its oxidative homeostasis in the absence of its symbiont [14,20]. Dependence phenotypes such as the one observed in *A*. *tabida* are the exception rather than the rule in arthropod–*Wolbachia* associations. However, subtler consequences of host adaptation to *Wolbachia* may be widespread and detectable in the form of “partial dependence”, *i*.*e*., a minor decrease in the host ability to withstand the absence of its symbiont. For example, the decrease in offspring productivity associated with the elimination of *Wolbachia* observed in some *Drosophila simulans* lines may be indicative of partial dependence [21].

In the present study, we looked for evidence of oxidative homeostasis-related partial dependence in *Asobara japonica*, a species closely related to *A*. *tabida*. In this species endemic from Japan, populations in the main islands are mostly composed of infected females reproducing asexually through *Wolbachia* induced thelytoky, whereas populations in southern islands are composed of uninfected males and females that reproduce sexually [22,23]. As *Wolbachia* disturbs the oxidative homeostasis in several species [4,6,12,18], we hypothesized that adaptation to *Wolbachia* in infected (thelytokous) populations of *A*. *japonica* involved modifications of oxidative homeostasis regulation processes. If so, these wasps may have evolved a form of partial dependence to *Wolbachia* that would manifest itself as a decreased ability to regulate the oxidative homeostasis in its absence. To test this hypothesis, we challenged the wasps with a pro-oxidant treatment (paraquat) and evaluated their sensitivity to this oxidative stress by measuring various life-history traits (parasitic success, larval development duration, body size, and potential fecundity). We measured oxidative stress sensitivity as the difference between the value of a trait in absence and in presence of the pro-oxidant treatment. We predicted that the sensitivity to oxidative stress should be higher in aposymbiotic *A*. *japonica* than in symbiotic ones. The reverse result, *i*.*e*., higher oxidative stress sensitivity in symbiotic wasps, may indicate that *Wolbachia* still disturbs the oxidative homeostasis of its host, despite the long-term relationship of the two partners. To control for potential direct effects of the antibiotic treatment used to obtain aposymbiotic individuals (*i*.*e*., effects unrelated to the elimination of *Wolbachia*), asymbiotic individuals from uninfected (arrhenotokous) populations were used as a control.

## Material & methods

### Biological material

*Asobara japonica* is a solitary parasitoid species of fruit flies. Four lines of *A*. *japonica* were used in the present study: two from thelytokous (infected) populations, T1 and T2 (originating from Kagoshima and Tokyo, respectively) and two from arrhenotokous (uninfected) populations, A1 and A2 (originating from Amami-oshima and Iriomote-jima, respectively). Wasps were trapped in the field [24] and provided by MT Kimura. These lines were maintained in the lab at 21°C on a *Wolbachia*-free line of *Drosophila melanogaster* originating from Sainte-Foy-lès-Lyon (France). The experiment described hereafter was performed at the same temperature, with the same *Drosophila* line used as a host.

### Antibiotic and paraquat treatments

An antibiotic treatment (rifampicin) was performed to eliminate *Wolbachia* when present (as described in [23]). A paraquat treatment (Sigma-Aldrich) was performed (as described in [8]) to induce oxidative stress in the wasps through the generation of intracellular ROS [25]. In thelytokous populations, *A*. *japonica* is dependent upon *Wolbachia* for sexual reproduction [23], preventing uninfected T1 and T2 lines to be established. The antibiotic treatments were thus performed together with the oxidative challenge, and arrhenotokous uninfected lines were included in the experimental design to control for the direct effects of the antibiotic treatment, apart from the elimination of *Wolbachia*. In summary, the experimental design consisted in 4 lines of wasps and 4 modalities of treatment: no treatment, antibiotics but no paraquat, paraquat but no antibiotics, and both antibiotics and paraquat. Therefore, 150 μL of either water or a 2% rifampicin solution were deposited on 1.5 g of fly medium [26], to which were further added 150 μL of either water or a 10 mM solution of paraquat. The concentration of paraquat was determined during a preliminary experiment to ensure that the dose would neither be lethal, nor biologically negligible for the fly larvae (S1 Fig). One hundred drosophila eggs were deposited on this medium, which was then inserted in an agar-containing vial, together with two female wasps, which were allowed to lay eggs for two days. As the parasitic success calculation requires a control of *Drosophila* hatching rate, vials identical to the 4 modalities of treatment but without wasps were also included. Ten replicates (vials) were performed for each of the 20 modalities.

### Life-history traits measurements

For each *A*. *japonica* line and experimental modality, the following traits were measured: parasitic success, larval development duration, tibia length (as a proxy for body size), and egg load at emergence (as a proxy for potential fecundity). The emerging insects were counted each day to calculate the parasitic success of the wasps, as well as their larval development duration. The parasitic success *ps* was calculated as in [27]: *ps = p*_*i*_ */ (d*_*m*_*-d*_*i*_*)*, with *p*_*i*_ the number of wasps that emerged from the vial i, *d*_*i*_ the number of flies that emerged from the vial i, and *d*_*m*_ the average number of flies that emerged from the control vials (without wasp) of the relevant treatment modality. The 5 vials in which no wasps emerged were removed from the analysis. The parasitic success was set to one when its calculated value was greater than one. *A*. *japonica* is mostly proovogenic, which means that females emerge with a stock of eggs, almost all of which are mature. To estimate potential fecundity, twenty females were isolated at emergence from the ten replicate vials of each modality, and fed with honey for five days, to ensure that egg maturation is complete and that their egg load is a reliable estimate of their potential fecundity. The two ovaries were dissected in a Phosphate Buffer Saline solution (1X PBS pH 7.4, Eurobio), individually transferred onto a slide, and then gently crushed between a slide and coverslip to disperse the egg content. These preparations were observed under a microscope (AxioCam Imager Z.1, Zeiss), and the eggs in both ovaries were counted using ImageJ software [28]. Egg load was calculated as the sum of eggs in the two ovaries. The length of the tibia of the left hind leg, was measured under the microscope on the same females, as a proxy for body size.

### Statistical analyses

A statistical model was fitted for each life-history trait. The parasitic success data were analysed by means of a mixed generalized linear model with a binomial distribution and a logit link function. The model was fitted with three fixed factors: reproductive mode (arrhenotokous or thelytokous), antibiotic treatment (yes or no) and paraquat treatment (yes or no) and their interactions. The line factor (A1, A2, T1 and T2) was included as a random effect. The overdispersion of the data was corrected using an observation-level random effect [29]. The larval development duration data were analysed by means of a mixed generalized linear model with a Gamma distribution and an inverse link function. The fixed effect factors were the same as those used in the model fitted for the parasitic success data. The line factor was also included as a random effect. The replicate vial factor was included as a random effect to account for the potential pseudo-replication due to the insects developing in the same vial. Body size (length of the tibia) and potential fecundity (egg load) were analysed by means of mixed linear models with the line factor as a random effect. Homoscedasticity and normality were checked graphically. In the body size model, the fixed effect factors were the same as in the two previously described models. In the potential fecundity model, by contrast, the length of the tibia was included as a quantitative explanatory variable to correct for the influence of body size on potential fecundity. For each model, we focused on the significance of the interaction between reproductive mode, antibiotic treatment and paraquat treatment, as well as the sign of this interaction. Indeed, we were interested in knowing whether the presence of *Wolbachia*–which, in thelytokous individuals, depends on whether an antibiotic treatment has been performed–has an impact on oxidative stress sensitivity, measured as the effect of the paraquat treatment. All statistical analyses were conducted using the R software version 3.2.4 [30].

## Results & discussion

Based on evidences coming from *A*. *tabida* [18–20] and other host species, such as mosquitoes [6,12] and flies [4], we hypothesized that coevolution between insects and *Wolbachia* generally results in the host reduced ability to regulate oxidative stress in the absence of its symbiont. We tested this hypothesis by comparing the effect of antibiotics on oxidative stress sensitivity in thelytokous (naturally infected) *versus* arrhenotokous (naturally uninfected) lines of the wasp *A*. *japonica*.

**Fig 1:**
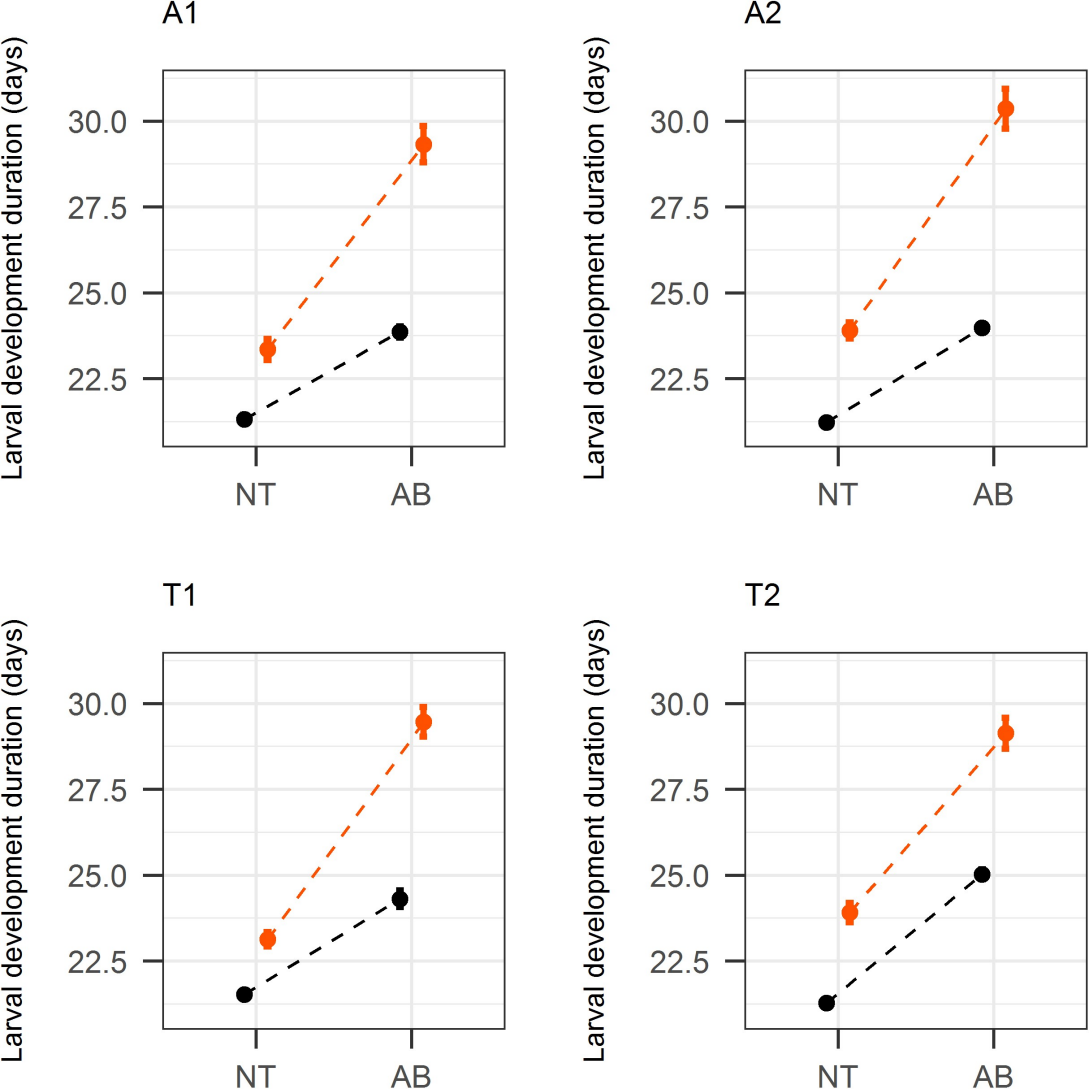
Larval development duration (mean ± SE) for each Asobara japonica line, with (AB) or without (NT) antibiotic treatment and with (orange) or without (black) paraquat.

**Fig 2:**
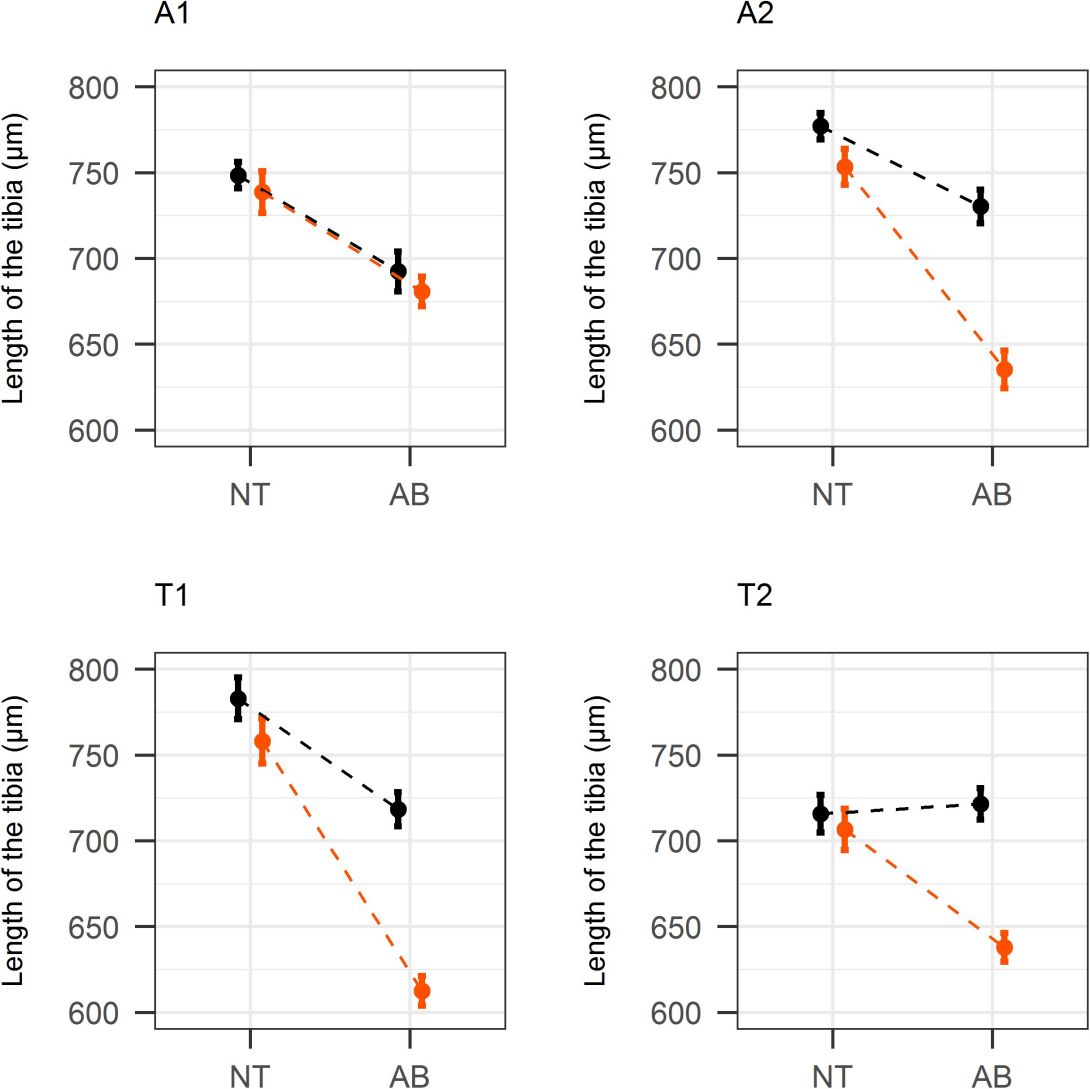
Tibia length (mean ± SE) for each Asobara japonica line, with (AB) or without (NT) antibiotic treatment and with (orange) or without (black) paraquat.

**Fig 3:**
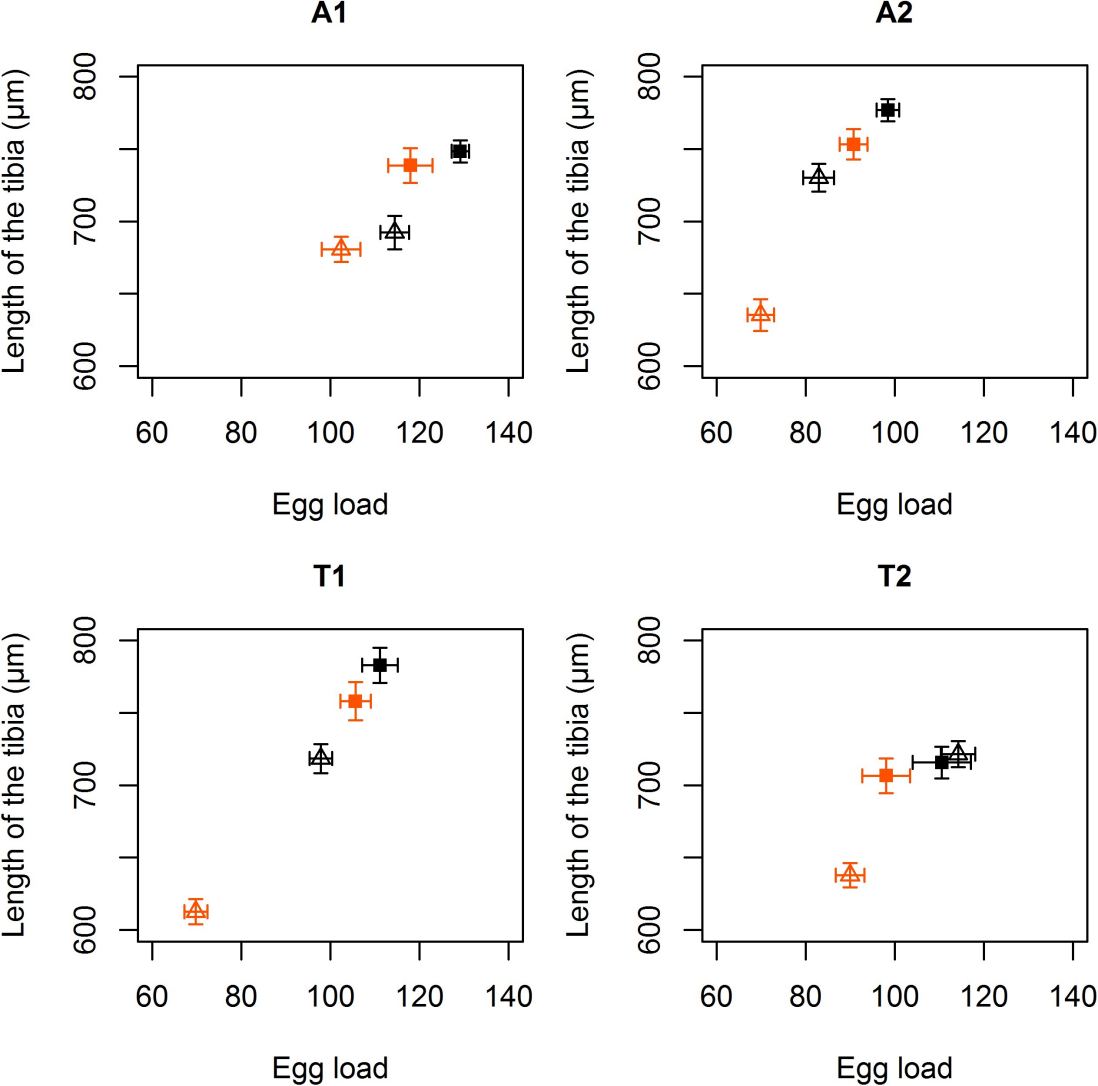
Egg load (mean ± SE) and tibia length (mean ± SE) for each Asobara japonica line, with (triangles) or without (squares) antibiotic treatment and with (orange) or without (black) paraquat.

Contrary to our expectation, we found that the impact of antibiotics on oxidative stress sensitivity was rarely associated with the infection status. Indeed, the interaction between reproductive mode, antibiotic treatment, and paraquat treatment was not statistically significant for larval development duration (χ^2^_1_ = 0.17, p = 0.68; Fig 1), body size (χ^2^_1_ = 3.68, p = 0.06; Fig 2), and potential fecundity (χ^2^_1_ = 0.25, p = 0.62; Fig 3). However, a statistically significant effect of the paraquat treatment was found on all these traits: larval development duration: χ^2^_2_ = 116.49, p<0.001; body size: χ^2^_2_ = 82.46, p<0.001; potential fecundity: χ^2^_1_ = 6.16, p = 0.01. Furthermore, the effect of the paraquat treatment is likely to be deleterious, as it leads to an increase of the larval development duration (in all cases; Fig 1), a decrease of the body size (in some experimental modalities; Fig 2), or a decrease of the potential fecundity (in some experimental modalities; Fig 3). These results indicate that the paraquat treatment was sufficient to induce an oxidative stress on the wasps.

**Fig 4:**
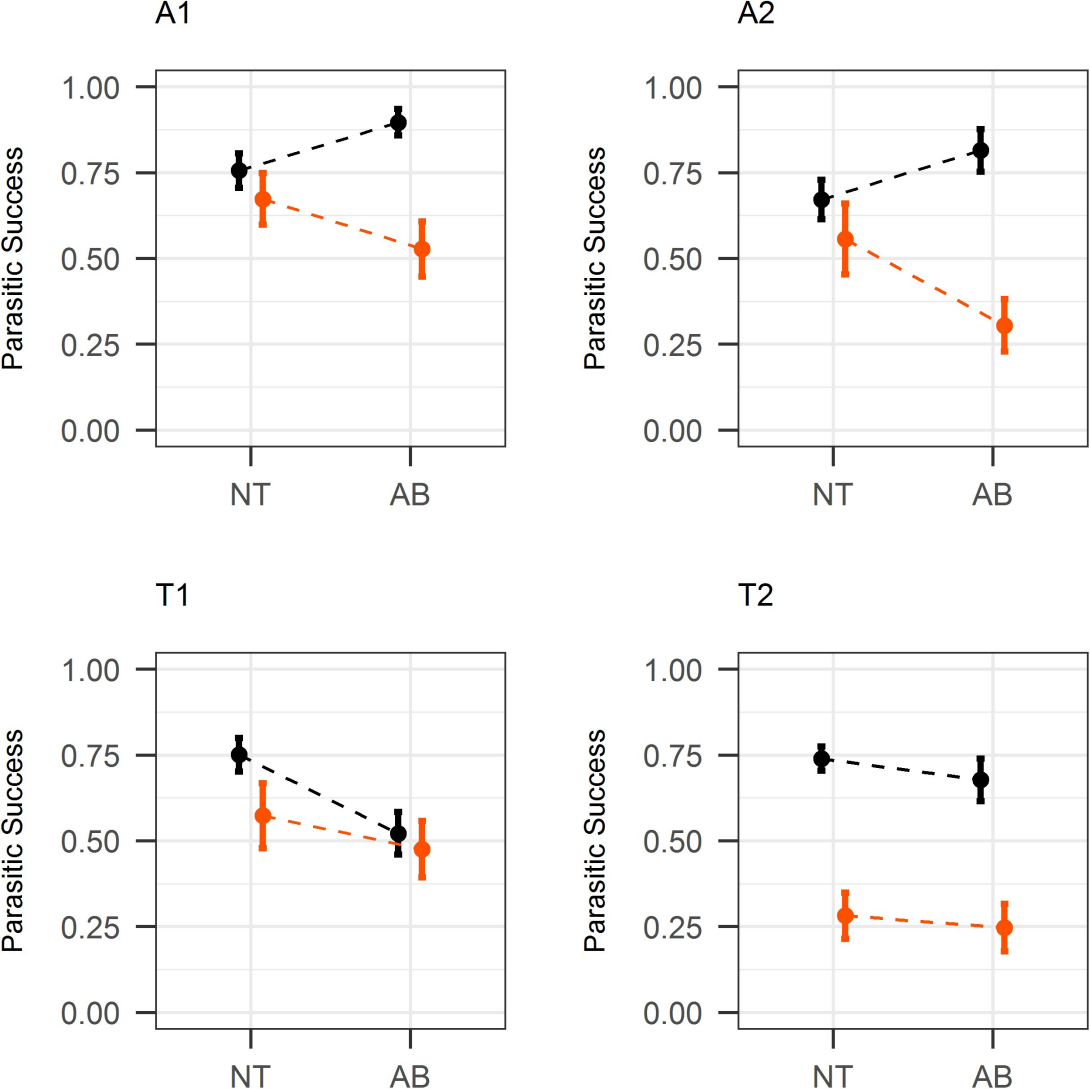
Parasitic success (mean ± SE) for each Asobara japonica line, with (AB) or without (NT) antibiotic treatment and with (orange) or without (black) paraquat.

The interaction between reproductive mode, antibiotic treatment, and paraquat treatment was statistically significant for parasitic success (χ^2^_1_ = 10.95, p<0.001; Fig 4). Indeed, the oxidative stress sensitivity was increased by the antibiotic treatment in the two arrhenotokous lines, but not in the two thelytokous lines. Although the paraquat treatment reduced the parasitic success of T2 but not of T1 wasps, these two lines were similar in that the antibiotic treatment did not increase their oxidative stress sensitivity.

In arrhenotokous lines, the antibiotic treatment increased parasitic success in the absence of the paraquat treatment (z = 3.19, p = 0.001; mean difference: 0.14±0.05). This positive effect of antibiotic treatment on parasitic success in absence of paraquat treatment was not observed in thelytokous lines (z = -1.77, p = 0.077). This suggests that the elimination of *Wolbachia* is detrimental to parasitic success in these conditions. This beneficial effect of *Wolbachia* possibly reflects a partial dependence of *A*. *japonica* upon it.

In arrhenotokous lines, the antibiotic treatment decreased parasitic success in the presence of the paraquat treatment (z = -2.59, p = 0.010; mean difference: 0.19 ± 0.09). This negative effect of the antibiotic treatment on parasitic success in the presence of the paraquat treatment was not observed in the thelytokous lines (z = -0.71, p = 0.478). It seems therefore that in thelytokous lines, the elimination of *Wolbachia* prevented the synergistic deleterious effect of the antibiotic and paraquat treatments observed in the arrhenotokous lines. Although *A*. *japonica* may not face oxidative stresses as intense as the one induced by the paraquat treatment in natural conditions, this result suggests that the elimination of *Wolbachia* increases the wasp ability to regulate oxidative stress. If so, the ability of *A*. *japonica* to control its oxidative homeostasis may still be impaired by the presence of *Wolbachia*, rather than being dependent upon it. We expect such a deleterious effect of *Wolbachia* to be eliminated in the course of host–symbiont coevolution. Its persistence may indicate that the infection by *Wolbachia* is either too recent, or not costly enough in natural conditions, for adaptation to have occurred.

The deleterious synergy between the antibiotic and paraquat treatments is a striking aspect of our results, as it is observed for most life-history traits and lines, including uninfected ones. Although a direct chemical interaction between the two compounds cannot be ruled out, another possibility is that the microbiota, apart from *Wolbachia* (*e*.*g*., the gut microbiota), is also altered by the antibiotic treatment and play an important role in oxidative homeostasis regulation.

Strictly speaking, our results do not rule out the possible dependence of *A*. *japonica* upon *Wolbachia* for the control of its oxidative homeostasis. Indeed, we focused only on the sensitivity to oxidative stress, *i*.*e*., to an excess of ROS. However, a deficit of ROS may also be a deleterious condition and it is therefore possible for the insect to evolve a dependence towards *Wolbachia* for the remediation of this condition. Replacing the pro-oxidant treatment (paraquat) with an antioxidant treatment (*e*.*g*., glutathione) in our experimental setup would allow testing the hypothesis that the elimination of *Wolbachia* aggravates the wasp sensitivity to a deficit of ROS.

However, our results suggest that *A*. *japonica* did not evolve a partial dependence to *w*Ajap for the regulation of oxidative stress. This result contrasts with what is observed in *A*. *tabida*, highlighting the need for a better understanding of the factors influencing host–*Wolbachia* coevolution. In particular, the assumption that the host is likely to adapt to its symbiont can be questioned. Indeed, the response to the infection may be plastic, and therefore reversible (not leading to any impairment if the symbiont is lost). Furthermore, even if genetic evolution occurs, it may occur in the symbiont rather than in the host (*e*.*g*., [31]), in which case the latter will not evolve any dependence.

## Supporting information

S1 Fig Impact of the dose of paraquat on the length of the wing in *Drosophila melanogaster*. 150 μL of each solution were added to 1.5 g of standard *Drosophila* medium, on which 100 eggs were deposited. The highest dose (100 mM) was lethal (no egg reached adulthood). In the three remaining modalities, the dose of paraquat had a statistically significant effect on the length of the wing (linear model, F_2,34_ = 7.76, p = 0.0017). The lowest dose (1 mM) had no effect on the length of the wing (Tukey post hoc test, adjusted p-value = 0.89) whereas the flies treated with the intermediary dose (10 mM) had shorter wing than both the untreated (Tukey post hoc test, adjusted p-value = 0.0079) and the treated with the lowest dose flies (Tukey post hoc test, adjusted p-value = 0.0028).
(TIF)

S1 File Supporting data. (XLSX)
