# Supplementary figures and images for: Impact of Wolbachia on oxidative stress sensitivity in the parasitic wasp Asobara japonica
Source: PLoS One. 2017 Apr 20;12(4):e0175974. doi: 10.1371/journal.pone.0175974 (PMC5398613; doi:10.1371/journal.pone.0175974)

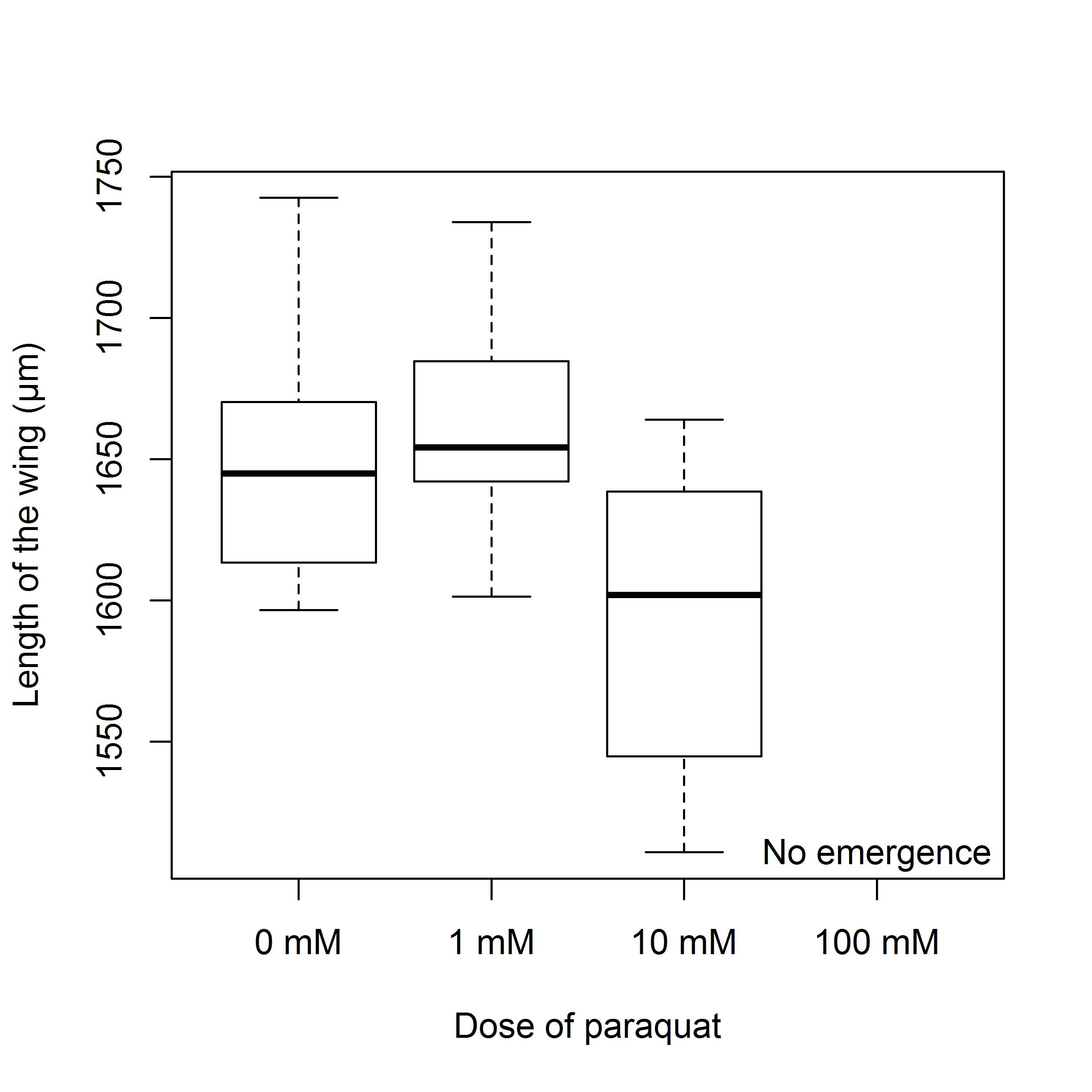

Supplement: S1 Fig — 150 μL of each solution were added to 1.5 g of standard Drosophila medium, on which 100 eggs were deposited. The highest dose (100 mM) was lethal (no egg reached adulthood). In the three remaining modalities, the dose of paraquat had a statistically significant effect on the length of the wing (linear model, F2,34 = 7.76, p = 0.0017). The lowest dose (1 mM) had no effect on the length of the wing (Tukey post hoc test, adjusted p-value = 0.89) whereas the flies treated with the intermediary dose (10 mM) had shorter wing than both the untreated (Tukey post hoc test, adjusted p-value = 0.0079) and the treated with the lowest dose flies (Tukey post hoc test, adjusted p-value = 0.0028). (TIF) [file pone.0175974.s001.tif]
